# 1329. Burden of Respiratory Syncytial Virus (RSV) Infection among Hospitalized Older Adults and Those with Underlying Chronic Obstructive Pulmonary Disease (COPD) or Congestive Heart Failure (CHF)

**DOI:** 10.1093/ofid/ofab466.1521

**Published:** 2021-12-04

**Authors:** Luis W Salazar, Ashley Tippett, Laila Hussaini, Megan Taylor, Olivia Reese, Caroline Ciric, Laurel Bristow, Vikash Patel, Wensheng Li, Hui-mien Hsiao, Kathy Stephens, Theda Gibson, Andrew Cheng, Ariel Kay, David L Swerdlow, Robin Hubler, Ben Lopman, Larry Anderson, Christina A Rostad, Nadine Rouphael, Nadine Rouphael, Evan J Anderson

**Affiliations:** 1 Emory University School of Medicine, Atlanta, Georgia; 2 Pfizer, Inc, New York, NY; 3 Pfizer Inc, Collegeville, PA; 4 Rollins School of Public Health, Emory University, Atlanta, GA; 5 Emory University, Atlanta, GA; 6 Emory University, Atlanta VA Medical Center, Atlanta, Georgia

## Abstract

**Background:**

The burden of Respiratory Syncytial Virus (RSV)-associated hospitalization in adults is incompletely understood. The COVID-19 pandemic has resulted in multiple public health measures (e.g., social distancing, handwashing, masking) to decrease SARS-CoV-2 transmission, which could impact RSV-associated hospitalizations. We sought to compare RSV-associated hospitalizations from 2 pre- and one mid-COVID-19 winter viral respiratory seasons.

**Methods:**

We conducted an IRB-approved prospective surveillance at two Atlanta-area hospitals during the winter respiratory viral seasons from Oct 2018–Apr 2021 for adults ≥ 50 years of age admitted with acute respiratory infections (ARI) and adults of any age with COPD or CHF-related admissions. Adults were eligible if they were residents of an 8 county region surrounding Atlanta, Georgia. Those with symptoms > 14 days were excluded. Standard of care test results were included. Asymptomatic adults ≥ 50 years of age were enrolled as controls in Seasons 1 and 2. Nasopharyngeal swabs from cases and controls were tested for RSV using BioFire® FilmArray® Respiratory Viral Panel (RVP). We compared the demographic features and outcomes of RSV+ cases and controls.

**Results:**

RSV was detected in 71/2,728 (2.6%) hospitalized adults with ARI, CHF, or COPD and 4/466 (0.9%) controls. In Season 1, RSV occurred in 5.9% (35/596 patients), in Season 2 3.6% (35/970 patients), but in only 0.09% (1/1,162 patients) in Season 3 (P < 0.001 for both seasons). RSV detection in Season 3 was similar to RSV detection among controls during Seasons 1 and 2 (P=0.6). Median age of cases and controls was 67 years (Table 1). Of cases with RSV 11% were admitted to the ICU and two required mechanical ventilation. The majority of hospitalized patients were discharged home (95.8%) with a median length of hospitalization of three days (IQR 2-7).

Table 1. Demographic Features and Outcomes Among RSV-Positive Hospitalized Adults.

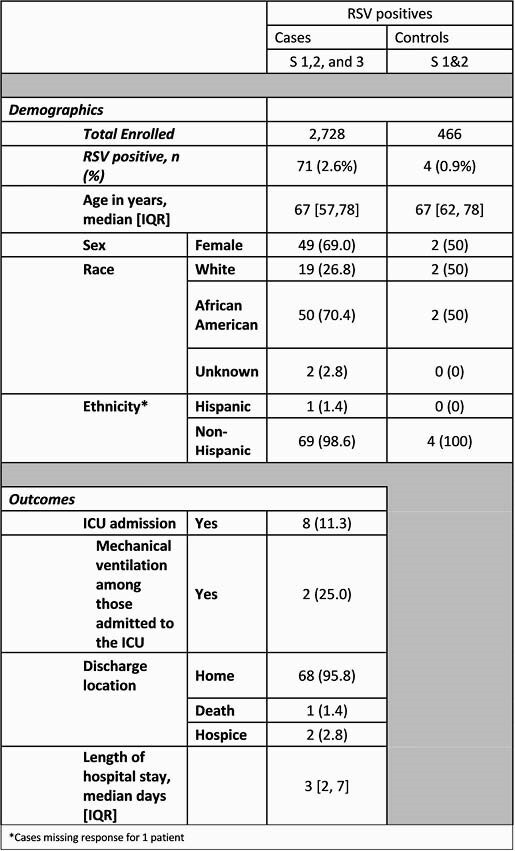

**Conclusion:**

Over 3 seasons, RSV was detected in 2.6% of adults admitted to the hospital with ARI, CHF or COPD. The rate of RSV dramatically declined during the 2020-21 winter respiratory viral season, likely due to public health measures implemented in response to COVID-19.

**Disclosures:**

**David L. Swerdlow, MD**, **Pfizer Vaccines** (Employee) **Robin Hubler, MS**, **Pfizer Inc.** (Employee) **Larry Anderson, MD**, **ADVI** (Consultant)**Bavarian Nordic** (Consultant)**Novavax** (Consultant)**Phizer** (Grant/Research Support, Scientific Research Study Investigator)**Sciogen** (Research Grant or Support) **Christina A. Rostad, MD**, **BioFire Inc, GSK, MedImmune, Micron, Janssen, Merck, Moderna, Novavax, PaxVax, Pfizer, Regeneron, Sanofi-Pasteur.** (Grant/Research Support, Scientific Research Study Investigator, Research Grant or Support)**Meissa Vaccines** (Other Financial or Material Support, Co-inventor of patented RSV vaccine technology unrelated to this manuscript, which has been licensed to Meissa Vaccines, Inc.) **Nadine Rouphael, MD**, **pfizer, sanofi, lily, quidel, merck** (Grant/Research Support) **Nadine Rouphael, MD**, Lilly (Individual(s) Involved: Self): Emory Study PI, Grant/Research Support; Merck (Individual(s) Involved: Self): Emory study PI, Grant/Research Support; Pfizer: I conduct as co-PI the RSV PFIZER study at Emory, Research Grant; Pfizer (Individual(s) Involved: Self): Grant/Research Support, I conduct as co-PI the RSV PFIZER study at Emory; Quidel (Individual(s) Involved: Self): Emory Study PI, Grant/Research Support; Sanofi Pasteur (Individual(s) Involved: Self): Chair phase 3 COVID vaccine, Grant/Research Support **Evan J. Anderson, MD**, **GSK** (Scientific Research Study Investigator)**Janssen** (Consultant, Scientific Research Study Investigator, Advisor or Review Panel member)**Kentucky Bioprocessing, Inc** (Advisor or Review Panel member)**MedImmune** (Scientific Research Study Investigator)**Medscape** (Consultant)**Merck** (Scientific Research Study Investigator)**Micron** (Scientific Research Study Investigator)**PaxVax** (Scientific Research Study Investigator)**Pfizer** (Consultant, Grant/Research Support, Scientific Research Study Investigator)**Regeneron** (Scientific Research Study Investigator)**Sanofi Pasteur** (Consultant, Scientific Research Study Investigator)

